# Chronotype and social jetlag influence human circadian clock gene expression

**DOI:** 10.1038/s41598-018-28616-2

**Published:** 2018-07-05

**Authors:** Masaki Takahashi, Yu Tahara, Miku Tsubosaka, Mayuko Fukazawa, Mamiho Ozaki, Tamao Iwakami, Takashi Nakaoka, Shigenobu Shibata

**Affiliations:** 1 Waseda Bioscience Research Institute in Singapore, Waseda University, Singapore, 138667 Singapore; 2 Department of Psychiatry and Biobehavioral Sciences, University of California, Los Angeles, 90024 USA; 3 Graduate School of Advanced Science and Engineering, Waseda University, Shinjuku, 162-8480 Japan; 4 Department of Medicine, Tokyo Women’s Medical University, Medical Center East, Arakawa, Tokyo, 116-8567 Japan; 5 Faculty of Science and Engineering, Waseda University, Shinjuku, 162-8480 Japan

## Abstract

We examined the relationships between chronotype or social jetlag and clock gene expression. Twenty-four young men [Chronotype: morningness, n = 8; intermediate, n = 8, eveningness, n = 8], aged 27 ± 2 years old (mean ± SE), completed two trials in a randomized order: (1) a Friday trial and (2) a Monday trial. In both trials, hair follicle cells were collected to evaluate the expression of clock genes over a 24-hour period at 4-hour intervals. There was a significant main effect of time on the expression of *NR1D1*, *NR1D2*, and *PER3* (P < 0.001) in the morningness group, but not in the eveningness group. Changes in the peak time of expression of *NR1D1* (r = 0.434, P = 0.034), *NR1D2* (r = 0.481, P = 0.017), and *PER3* (r = 0.457, P = 0.025) from the Friday to Monday trials were positively correlated with social jetlag (SJL) time. Our findings indicate that there was no change in the patterns of clock gene expression between workdays and the day after the holiday in the morningness group, and that SJL time influences the peak time of clock gene expression, moving it from the early to late workday, after a holiday.

## Introduction

Circadian rhythms have been shown to regulate several physiological functions, including body temperature, sleep, physical activity, mood, and cognition^1–3^. These processes are controlled by circadian clock genes, and abnormal circadian rhythms are associated with the development of obesity and lifestyle-related diseases^4–6^. In addition, the timing of behaviours such as sleep, exercise, and food intake influence circadian rhythms, including clock gene expression in peripheral tissues^7–10^.

Chronotype is an expression of individual circadian rhythmicity, which is related to sleep, diet, and physical activity patterns, including exercise^11–13^. Chronotype can be classified into three different types, namely, morningness, intermediate, and eveningness. Previous studies have shown that eveningness can be detrimental to sleep quality, as assessed by the Pittsburgh sleep quality index^14^. Another study has reported that individuals with an eveningness type have a significantly greater body mass index (BMI) compared with morningness and intermediate types^15^. In addition, it has been reported that eveningness is independently associated with lack of glycaemic control in type 2 diabetes^16^. Therefore, it is important to consider an individual’s chronotype when examining the association between lifestyle habits, including activity and sleep, and circadian rhythms.

Social jetlag (SJL), assessed by the difference in hours between the midpoint of sleep on weekdays and weekends, quantifies the discrepancy between biological rhythms and social timing^17,18^. Previous studies have shown a strong association between SJL and chronotype, with a later chronotype being associated with greater SJL^19^. Importantly, SJL is associated with obesity, diabetes, depression, and reduced academic performance^20–24^. Studies on the physiological mechanisms underlying SJL indicate that it is associated with an adverse endocrine, behavioural, and cardiovascular risk profile^25,26^. Although evidence related to the effects of SJL has increased, most studies have tended to use questionnaires to examine the relationship between SJL and health parameters. Moreover, previous studies have reported that clock gene dysfunction accelerates the development of several diseases, such as obesity, diabetes, and fatty liver diseases, and that these disorders also disrupt clock function^5,6,27^. From the viewpoint of elucidating physiological mechanisms and preventing various diseases, it may be important to examine the relationship between chronotype/SJL and circadian rhythms, including clock gene expression.

In recent years, several studies have reported that hair follicle cells can be used as a rapid and convenient means for evaluating the expression of human circadian clock genes^28–30^. This method has been developed to obtain peripheral tissues in a minimally invasive manner. Moreover, Akashi *et al*. have reported that circadian fluctuations in *PER3*, *NR1D1*, and *NR1D2* mRNA in hair follicle cells could be used as a more effective tool compared with other methods, such as those involving oral mucosa tissue, to evaluate circadian rhythms in humans^28,30^. In addition, it has been shown that the peak time of the expression of these clock genes is correlated with the time of waking in individuals^28^. However, little is known about the relationship between SJL and clock gene expression. Regardless of circadian control, we often use social clocks to align the time of sleeping and waking with social obligations such as school, work, and other social events^20^. Previous studies have indicated that Monday is more influenced by weekends for those who have a holiday on the weekend, and SJL can be used to assess this effect^17,20^. In the present study, we recruited participants who worked from Monday to Friday and had a day off on Sunday. We thus considered Friday to be a day with socially adapted rhythms (i.e. control day) and Monday as a day with socially regulated rhythms (i.e. SJL day). To elucidate the relationship between socially adapted and socially regulated rhythms, we compared clock gene expression in the Friday trial with that in the Monday trial. The purpose of the present study was to investigate the relationships between chronotype/SJL and clock gene expression. We hypothesized that changes in the human circadian system from workdays to after a holiday, as assessed by clock gene expression, would be strongly influenced by the SJL time of individuals.

## Results

### Physical characteristics of participants, chronotype groups, and high and low SJL groups

Data for the physical characteristics, MEQ score, sleep timing, and SJL time of all participants and participants in each chronotype group are shown in Table 1. There were no significant differences in age, height, body weight, or BMI among the individuals with different chronotypes. The MEQ score was significantly higher in the morningness group than in the intermediate (P = 0.001) and eveningness (P = 0.001) groups. There was also a significant difference in MEQ score between the intermediate and eveningness (P = 0.001) groups. The time of waking on weekdays was significantly delayed in the eveningness group compared with the morningness group (P = 0.003). The time of waking at weekends was also significantly delayed in the intermediate (P = 0.002) and eveningness (P = 0.001) groups compared with the morningness group. Bedtime on weekdays was significantly delayed in the eveningness group (P = 0.020) compared with the morningness group. Moreover, bedtime on weekends was significantly delayed in the intermediate (P = 0.009) and eveningness (P = 0.001) groups compared with the morningness group. SJL time values were significantly higher in the intermediate (P = 0.009) and eveningness (P = 0.003) groups compared with the morningness group.

**Table 1:** Physical characteristics of the all participants and each chronotype group.

	All (n = 24)	Morningness (n = 8)	Intermediate (n = 8)	Eveningness (n = 8)	P value
Age (years)	26.5 ± 0.9	27.9 ± 1.3	25.9 ± 2.3	25.1 ± 1.2	0.440
Height (cm)	172.8 ± 1.0	173.7 ± 2.3	173.4 ± 1.9	171.0 ± 1.6	0.465
Weight (kg)	68.1 ± 1.7	66.9 ± 2.1	71.4 ± 4.5	64.9 ± 1.5	0.169
BMI	22.7 ± 0.4	22.2 ± 0.6	23.8 ± 1.2	22.2 ± 0.6	0.199
MEQ score	49.5 ± 2.6	63.3 ± 1.3	50.1 ± 2.1**	35.3 ± 2.2**^,##^	0.001
Waking time at weekday (h)	7.4 ± 0.3	6.4 ± 0.3	7.6 ± 0.6	8.3 ± 0.2**	0.004
Bedtime at weekday (h)	24.3 ± 0.3	23.4 ± 0.5	24.5 ± 0.5	25.0 ± 0.2*	0.020
Waking time at weekend (h)	9.0 ± 0.4	7.0 ± 0.6	9.6 ± 0.5**	10.5 ± 0.5**	0.001
Bedtime at weekend (h)	24.9 ± 0.3	23.6 ± 0.4	25.3 ± 0.4**	25.9 ± 0.4**	0.001
Social jetlag time (h)	1.1 ± 0.2	0.4 ± 0.2	1.4 ± 0.3**	1.5 ± 0.2**	0.002

All data are presented as means ± SE.

P value: One-way ANOVA; among groups, *P < 0.05, **P < 0.01 vs Morningness; ^##^P < 0.01 vs Intermediate.

Data for the physical characteristics, MEQ score, sleep timing, and SJL time of participants in the small and large SJL groups are shown in Table S1. The SJL time in the high SJL group was significantly higher than that in the low SJL group (P = 0.001). There was no significant difference between the two groups in terms of age, height, body weight, BMI, and time of waking on weekdays and bedtime time at weekends. The MEQ score in the low SJL group was significantly higher than that in the high SJL group (P = 0.004). The time of waking and bedtime on weekends was significantly delayed in the high SJL group compared with that in the low SJL group (P = 0.011 and P = 0.001, respectively).

### Sleep and diet time of all participants and chronotype groups in Friday and Monday trials

Data for the sleep and diet time of all participants and chronotype groups in both trials are shown in Table 2. There were no significant differences in wake-up time and bedtime in the Friday trial. In contrast, there were significant differences among groups in wake-up time (P = 0.040) and bedtime (P = 0.002) in the Monday trial.

**Table 2:** Sleep and meal times of all participants and chronotype groups in Friday and Monday trials.

	All (n = 24)	Morningness (n = 8)	Intermediate (n = 8)	Eveningness (n = 8)	P value
**Friday trials**					
Wake-up time (h)	6.5 ± 0.3	6.2 ± 0.3	6.0 ± 0.1	7.4 ± 0.9	0.136
Bedtime (h)	24.9 ± 0.3	24.0 ± 0.6	25.3 ± 0.6	25.3 ± 0.6	0.184
Breakfast (h)	8.1 ± 0.2	7.1 ± 0.3	8.6 ± 0.4*	8.7 ± 0.3*	0.012
Lunch (h)	12.6 ± 0.2	11.9 ± 0.3	12.9 ± 0.4	13.0 ± 0.3	0.055
Dinner (h)	18.9 ± 0.7	17.6 ± 1.4	17.9 ± 1.7	21.1 ± 0.7*	0.025
**Monday trials**					
Wake-up time (h)	6.7 ± 0.3	6.4 ± 0.3	6.1 ± 0.3	7.8 ± 0.8^#^	0.040
Bedtime (h)	25.2 ± 0.3	23.7 ± 0.6	25.4 ± 0.6*	26.3 ± 0.3*	0.002
Breakfast (h)	7.7 ± 0.3	7.5 ± 0.5	8.1 ± 0.5	7.2 ± 0.4	0.278
Lunch (h)	12.7 ± 0.2	12.1 ± 0.3	12.7 ± 0.2	13.3 ± 0.5*	0.050
Dinner (h)	20.5 ± 0.3	19.4 ± 0.4	20.1 ± 0.3	22.1 ± 0.6*#	0.001

All data are presented as means ± SE.

P value: One-way ANOVA; among groups, *P < 0.05 vs Morningness; ^#^P < 0.05 vs Intermediate.

There were significant differences among groups in the time of breakfast (P = 0.012) and dinner (P = 0.025), and the time of lunch also tended to be different (P = 0.055) in the Friday trial. Time of breakfast and dinner was also significantly delayed in the eveningness group compared to the morningness (respectively; P = 0.015, P = 0.019) and intermediate groups (respectively; P = 0.042, P = 0.025). Moreover, there were significant differences among the groups in time of lunch (P = 0.049) and dinner (P = 0.001) in the Monday trial. Time of dinner was significantly delayed in the eveningness group compared to the morningness (P = 0.001) and intermediate groups (P = 0.040).

### Diurnal expression of clock genes in Friday and Monday trials in all participants

**Figure 1:**
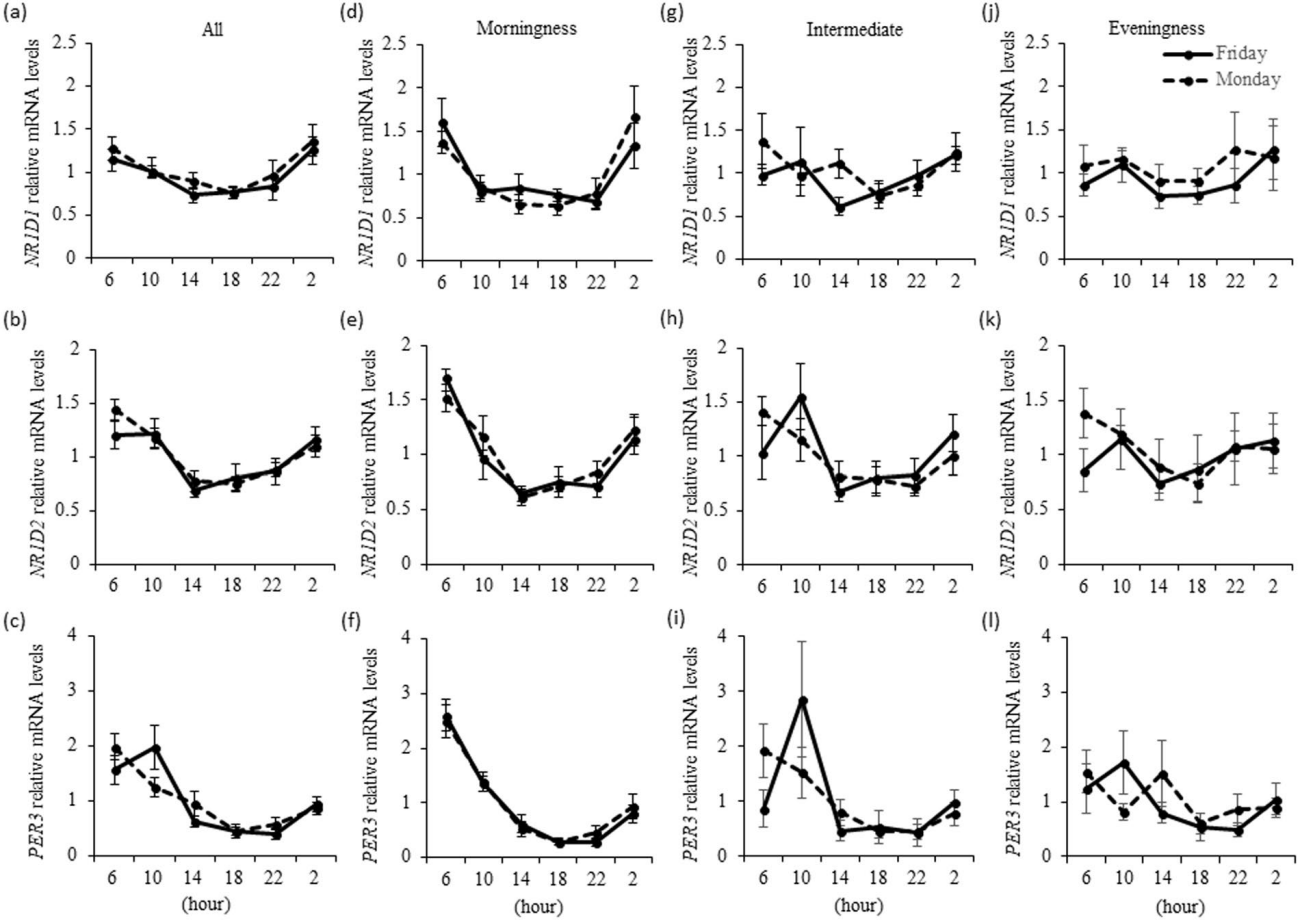
Diurnal expression of *NR1D1* (**a**) *NR1D2* (**b**) and *PER3* (**c**) genes in the hair follicle cells collected from all study participants (n = 24), and the diurnal expression of *NR1D1* (**d**,**g**,**j**) *NR1D2* (**e**,**h**,**k**) *PER3* (**f**,**i**,**l**) in the hair follicle cells of individuals in each chronotype group (morningness: n = 8, intermediate: n = 8, eveningness: n = 8). Data are the mean ± SE. There was a significant time effect (P = 0.001) observed for *NR1D1*, *NR1D2*, and *PER3* expression, and the time × trial interaction was also significant (P = 0.027) for *PER3* expression (two-way repeated measures ANOVA) in all participants. There was a significant time effect (P = 0.001) observed for *NR1D1*, *NR1D2*, and *PER3* expression in the morningness group, and for *NR1D2* (P = 0.004) and *PER3* (P = 0.001) expression in the intermediate group (two-way repeated measures ANOVA).

The patterns of *NR1D1*, *NR1D2*, and *PER3* genes expression in all participants in both trials were investigated in this study. Two-way ANOVA revealed a significant main effect of time for *NR1D1*, *NR1D2*, and *PER3* expression in all participants (P = 0.001 for each) [Fig. 1(a–c)]. Furthermore, we detected a significant trial × time interaction (P = 0.027) for *PER3* expression in all participants.

In the present study, most clock gene expression patterns could not be fitted by 24 h cosine rhythm analysis. Therefore, we defined the highest level of clock gene expression throughout whole day as the peak time of the clock system. The peak times of expression of clock genes in all participants are presented in Table 3. There were no significant differences in the peak times of all clock genes between the two trials.

**Table 3:** Peak time and change in peak time of clock gene expression in all participants and each chronotype.

		All	Morningness	Intermediate	Eveningness
*NR1D1*	Friday	7.03 ± 1.22	5.50 ± 0.91	8.50 ± 2.72	7.50 ± 2.38
	Monday	8.67 ± 1.27	5.00 ± 1.00	10.50 ± 1.92	10.50 ± 2.87
	Δ	4.50 ± 0.84	1.50 ± 0.73	5.00 ± 1.81	7.00 ± 1.00*
*NR1D2*	Friday	9.00 ± 1.06	5.50 ± 0.50	10.00 ± 1.69	11.50 ± 2.26
	Monday	8.00 ± 1.05	5.50 ± 0.91	7.50 ± 0.73	11.00 ± 2.70
	Δ	2.67 ± 0.67	1.00 ± 0.65	4.50 ± 1.40	2.50 ± 1.05
*PER3*	Friday	8.17 ± 0.90	6.00 ± 0.01	8.50 ± 1.05	8.50 ± 1.99
	Monday	8.83 ± 1.04	6.00 ± 0.76	5.50 ± 1.30	12.00 ± 2.51*
	Δ	3.67 ± 0.76	1.00 ± 0.65	1.41 ± 0.25	4.50 ± 1.40*

All data are presented as means ± SE. Δ = average of individual changes from Friday to Monday trials.

P value: Bonferroni test; among groups, *P < 0.05 vs Morningness group.

### Diurnal expression of clock genes in each chronotype group

The results for each chronotype group were examined by two-way ANOVA. As shown in Fig. 1, there was a significant main effect of time on the expression of *NR1D1*, *NR1D2*, and *PER3* (P = 0.001 for each) in the morningness group [Fig. 1(d–f)] and intermediate group [Fig. 1(g–i)], excluding *NR1D1*, but not in the eveningness group [Fig. 1(j–l)]. The results of peak time of expression of clock gene in each chronotype are in Table 3. In the eveningness group, the peak time of expression for *PER3* was delayed compared with that in the morningness group (P = 0.050). In addition, the change in the peak time of *NR1D1* (P = 0.018) and *PER3* (P = 0.038) expression from the Friday to Monday trial was higher in the eveningness group than in the morningness group (Table 3).

### Diurnal expression of clock genes in low and high SJL groups in Monday and Friday trials

**Figure 2:**
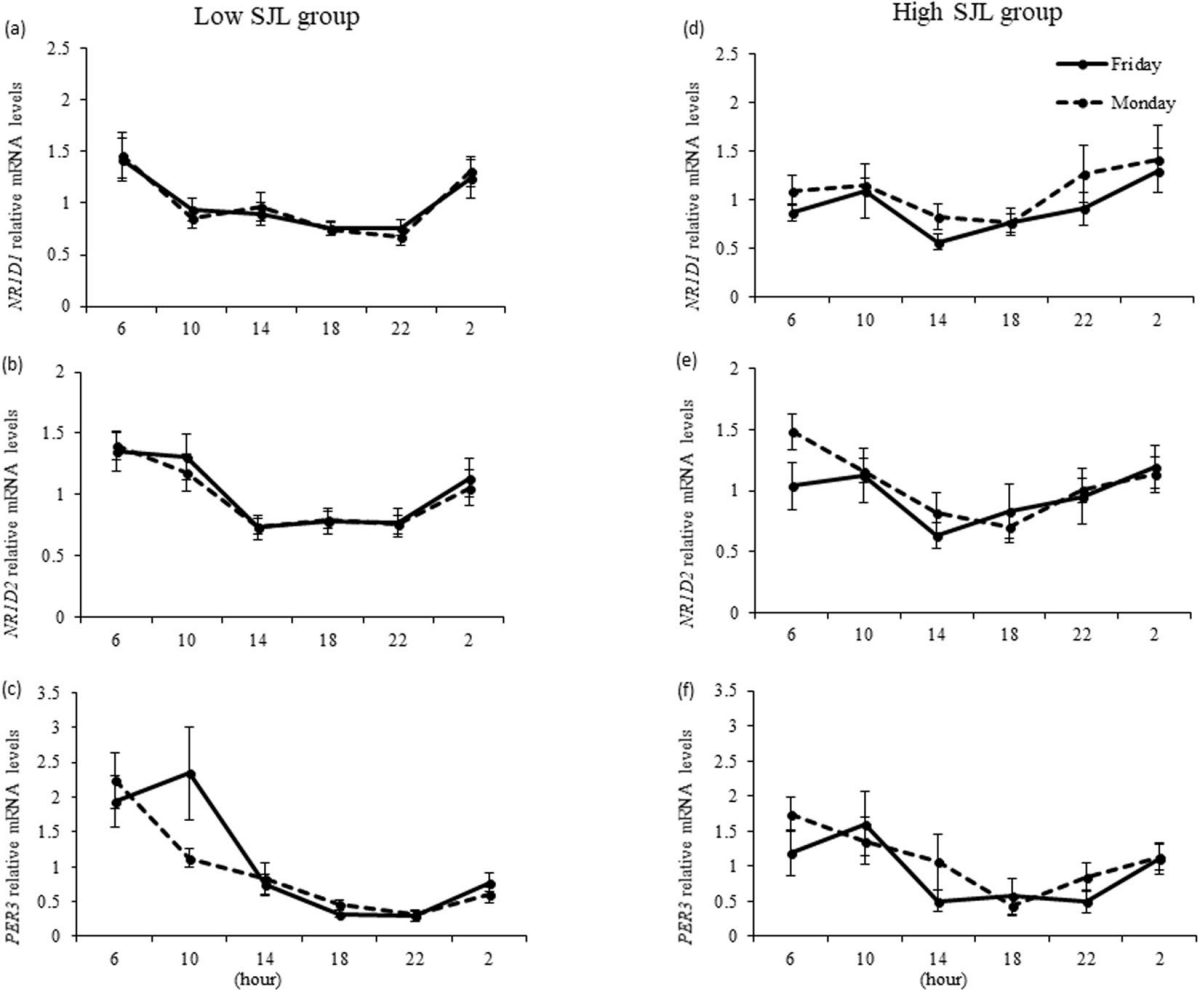
Diurnal expression of *NR1D1* (**a**,**d**) *NR1D2* (**b**,**e**) and *PER3* (**c**,**f**) in hair follicle cells of individuals in the low social jetlag (SJL) (n = 12) and high SJL (n = 12) groups. Data are the mean ± SE. A significant time effect (P = 0.001) was observed for *NR1D1*, *NR1D2*, and *PER3* expression in both groups.

Two-way ANOVA revealed a significant main effect of time on *NR1D1*, *NR1D2*, and *PER3* expression (P = 0.001 for each) in both the low and high SJL groups [Fig. 2(a–f)]. The peak times of the expression of clock genes in both groups are presented in Table 4. There was no significant difference in the peak time between the two trials in either group. Moreover, there was no significant difference in the change in peak time from the Friday to Monday trial in either group (Table 4).

**Table 4:** Peak time and change in peak time of clock gene expression in low and high SJL groups.

		Low SJL group	High SJL group
*NR1D1*	Friday	5.67 ± 0.77	8.67 ± 2.27
	Monday	6.67 ± 1.19	10.67 ± 2.14
	Δ	3.67 ± 1.15	5.33 ± 1.24
*NR1D2*	Friday	7.67 ± 0.92	10.33 ± 1.87
	Monday	6.67 ± 0.67	9.33 ± 1.96
	Δ	1.67 ± 0.59	3.67 ± 1.15
*PER3*	Friday	7.67 ± 0.77	8.67 ± 1.66
	Monday	7.67 ± 0.92	10.00 ± 1.84
	Δ	2.67 ± 0.90	4.67 ± 1.19

All data are presented as means ± SE. Δ = average of individual changes from Friday to Monday trials.

### Relationships between peak time of clock gene expression and MEQ

The peak time of *NR1D2* expression in the Friday trial was negatively correlated with MEQ score (*NR1D2*: r = −0.561, P = 0.004). In addition, the peak time of *NR1D1*, *NR1D2*, and *PER3* expression in the Monday trial was negatively correlated with MEQ score (*NR1D1*: r = −0.434, P = 0.034, *NR1D2*: r = 0.534, P = 0.007; *PER3*: r = −0.573, P = 0.003) (Table 5).

**Table 5:** Correlations between peak time of clock gene expression and MEQ score in all participants.

MEQ		NR1D1	NR1D2	PER3
Friday	r	−0.007	−0.561	−0.375
	P	0.976	0.004	0.071
Monday	r	−0.434	−0.534	−0.537
	P	0.034	0.007	0.003

*r*, correlation coefficient; *P*, P value.

### Relationship between changes in the peak time of clock gene expression and SJL time

**Figure 3:**
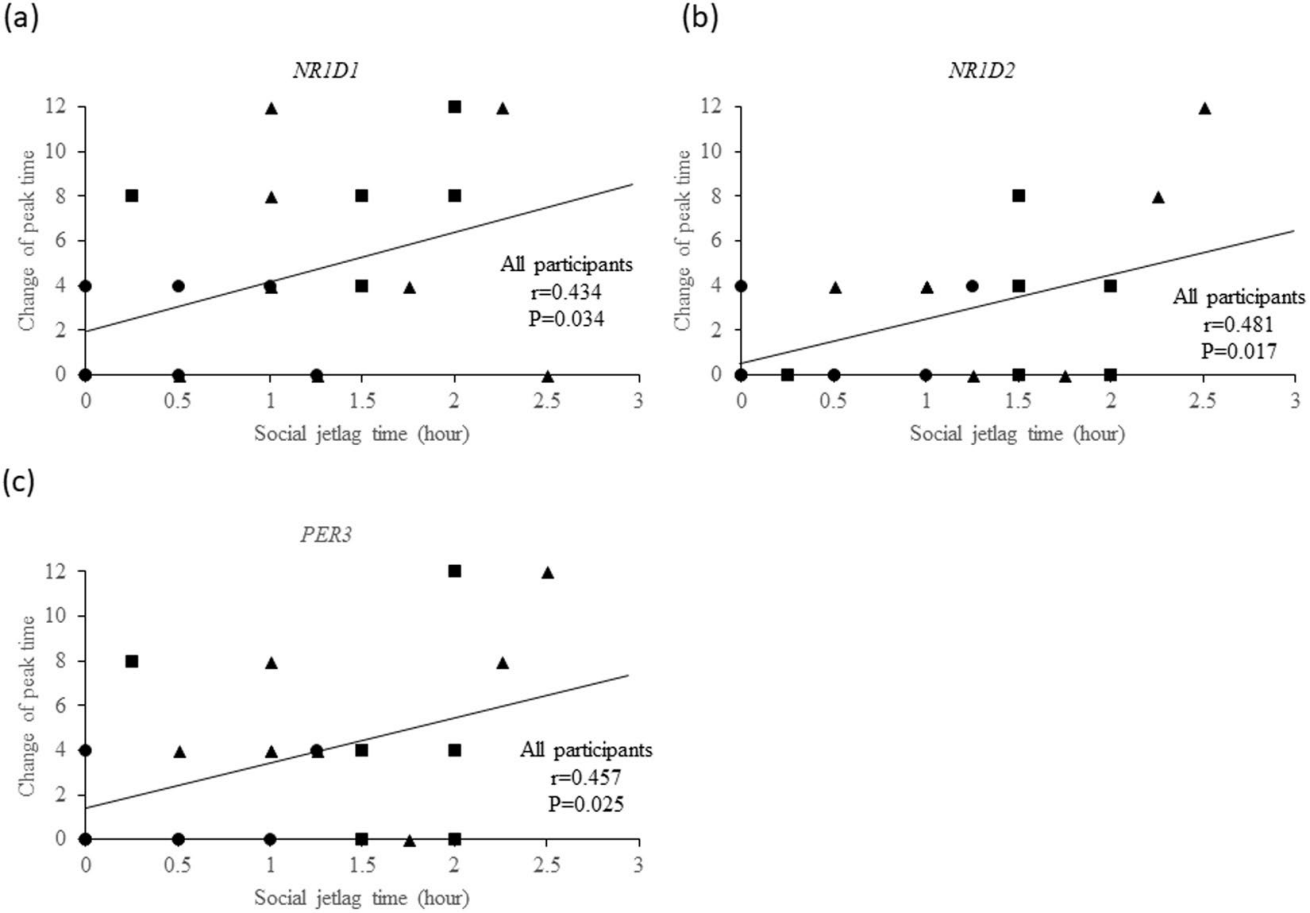
Relationship between changes in peak time for the expression of each clock gene [*NR1D1* (**a**) *NR1D2* (**b**) *PER3* (**c**)] and social jetlag time (n = 24) in Friday and Monday trials [Pearson’s correlation coefficients: (**a**) P = 0.034, (**b**) P = 0.017, (**c**) P = 0.025]. The change in peak time was calculated as an absolute value.

Changes in the peak time of *NR1D1*, *NR1D2*, and *PER3* expression between the Friday and Monday trials were positively correlated with SJL time (*NR1D1*: r = 0.434, P = 0.034; *NR1D2*: r = 0.481, P = 0.017; *PER3*: r = 0.457, P = 0.025) [Fig. 3(a–c)]. These data suggest that SJL time in individuals had an influence on the peak time of the circadian rhythm.

### Relationships between peak time of clock gene expression and mealtime

The peak time of *NR1D2* expression in the Friday trial was positively correlated with dinner time (*NR1D2*: r = 0.469, P = 0.021) (Table S2). The peak time of *NR1D2* and *PER3* expression in the Monday trial was positively correlated with dinner time (*NR1D2*: r = 0.536, P = 0.008; *PER3*: r = 0.576, P = 0.004).

## Discussion

To the best of our knowledge, the present study is the first to examine the relationships between chronotype/SJL and the expression of clock genes in humans. The main finding was that there was no change in the patterns of clock gene expression between workdays and the day after the holiday in the morningness group, whereas expression patterns did change in the eveningness group. In addition, changes in the peak time of clock gene expression were positively correlated with SJL time. These findings indicate that chronotype and SJL may affect the internal circadian clock system. Notably, SJL time in individuals is correlated with the change in peak clock gene expression time between workdays and the day after a holiday.

Several studies have shown that evaluating peripheral clock gene expression in hair follicle cells could be an effective method for assessing human circadian rhythms^7,28,29^. Indeed, in our previous study, we demonstrated that examining clock gene expression using hair follicle cells is also an effective tool in older adults^31^. In the present study, we compared the expression of clock genes between workdays and the day after a holiday in young adults. Regardless of circadian control, social clocks are often used to align the sleep and waking times of individuals with social obligations such as school, work, and other social events^20^. Our study showed that the expression of these genes changed according to the time of day in all participants. Moreover, as shown by patterns of *PER3* expression, there were significant differences between trials in all participants. These findings indicate that *PER3* expression differs between school or workdays and the day after a holiday.

It is recognized that chronotype reflects an individual’s circadian preferences in the timing of sleep and is also determined by other factors such as genetics, age, sex, and environment^32–34^. Compared with morningness individuals, eveningness people prefer a later bedtime and waking time. In the present study, the average bedtime and waking time was approximately 0:13 AM and 7:28 AM, respectively, on weekdays and approximately 0:54 AM and 9:00 AM, respectively, during the weekend. A comparison among chronotypes indicated that bedtime and waking time tended to be delayed in the eveningness group compared with those in the morningness group. Although the characteristics of the participants in each chronotype group were similar to those examined in previous studies, there remains uncertainty regarding the relationship between chronotype and the internal circadian clock system. Interestingly, our results showed significant time effects on the expression of *NR1D1*, *NR1D2*, and *PER3* in the morningness group but not in the eveningness group. These findings indicate that chronotype may influence the patterns of clock gene expression. A previous study reported that the circadian phases of clock gene expression in the oral mucosa were delayed in eveningness chronotypes compared with those in morningness chronotypes under real-life conditions^35^. Another study showed that compared to extreme morningness chronotypes, extreme eveningness chronotypes have a delay in the phase of circadian clock gene expression in hair follicle cells^36^. In the present study, the peak time of *PER3* expression was delayed in the eveningness group compared with that in the morningness group. In addition, the peak times of the expression of all clock genes were negatively correlated with MEQ score in the Monday trial, but only the peak time of *NR1D2* expression was correlated with MEQ score in the Friday trial. These results indicate that the chronotype of individuals shows a greater correlation with the peak time of circadian rhythms on the day after a holiday.

SJL represents the discrepancy between internal circadian clocks and social clocks^20,26^. A previous study showed that one-third of the population suffers from 2 hours or more of SJL, and approximately two-thirds of a European population (from younger to older generations) reported at least 1 hour of SJL^20^. Another study showed that, among individuals with non-communicable chronic diseases, SJL (>1 hour) was associated with a higher risk of being overweight and suffering from related metabolic complications^23^. With reference to previous studies, we calculated the SJL time in the present study by subtracting the midpoint of sleep on a work or school day from that on a free day^17,20,23^. The results of the present study show that the average SJL time for all participants was approximately 1 hour. There were, however, significant differences in SJL time among the chronotype groups, with the intermediate (average 1.4 ± 0.2 hours) and eveningness (average 1.5 ± 0.2 hours) groups showing longer SJL times than the morningness group (average 0.4 ± 0.2 hours). Notably, changes in the peak time of *NR1D1*, *NR1D2*, and *PER3* expression between the Friday and Monday trials were positively correlated with SJL time. Although there was a significant time effect on the expression of all three clock genes, no significant differences in clock gene expression patterns were observed between the small and large SJL groups. Therefore, SJL time may influence the change in peak clock gene expression time from workdays (i.e. Friday) to the day after a holiday (i.e. Monday), as assessed by clock gene expression in young adults.

The present study does, nonetheless, have some limitations. First, no data were obtained to assess the expression patterns of clock genes on a holiday (i.e. Sunday). Thus, changes in the expression of clock genes on weekends, including Saturdays, remain undetermined. Second, it is unclear whether other external synchronizers, particularly light, had an effect on clock gene expression in this study. In general, participants in the morningness group may experience more natural light time, whereas those in the eveningness group may spend more time under artificial light. Moreover, this study lacked data regarding physical activity patterns. Some studies have shown that high-intensity exercise influences the expression of clock genes^7,9^. Although further investigations are needed to consider the effect of light exposure or physical activity patterns on clock gene expression, the present investigation is valuable with respect to obtaining important findings in field experiments rather than in laboratory experiments. Another entrainment factor is mealtime, which may strongly adjust or regulate circadian rhythms^10,37,38^. In our study, the peak time of *NR1D2* expression in both trials and *PER3* expression in the Monday trial was positively correlated with dinner time (Table S2). Although we did not evaluate mealtimes in daily life, we asked each subject to maintain normal eating habits during the day before sampling and on the day of sampling itself. Thus, we assume that the mealtimes on the experimental days were reflected in the daily mealtimes. Finally, SJL, chronotype, and meal timing may be confounding factors. Although the use of multiple linear regression was not possible in this study because of the small sample size and the lack of daily mealtime data, additional research in humans with larger sample sizes should be performed to assess differences among SJL, chronotype, and daily mealtime or diet content and clock gene expression patterns.

## Conclusions

In conclusion, our study has revealed that chronotype and SJL are closely related to the rhythm of clock gene expression in young adults and that SJL time in particular influences the change in the peak time of clock gene expression from workdays to the day after a holiday in individuals.

## Methods

### Participants

Twenty-four healthy men participated in this study after giving written informed consent. None of the participants was involved in athletic activities, although some were recreationally active. In addition, all participants worked from Monday to Friday and had a day off on Sunday. The exclusion criteria included yielding an insufficient total amount of RNA and taking psychotropic, sleep, steroid, diabetes, or hyperlipidaemia related medicines. In addition, individuals who were obese (BMI > 30 kg/m^2^), or who suffered from sleep apnea syndrome or high blood pressure (systolic blood pressure >140 mmHg, diastolic blood pressure >90 mmHg), were excluded. The physical characteristics of the participants are shown in Table 1. This study was conducted according to the guidelines laid down in the Declaration of Helsinki and was approved by the ethics committees of Waseda University (2015–089). Informed consent was obtained from all participants following a detailed description of the experiment.

### Experimental protocol

Under daily living conditions, each participant completed two trials in a randomized order: (1) a Friday trial and (2) a Monday trial within 1 week. Sunday was a holiday, and Monday to Friday were workdays for all participants. Hair follicle cells were collected from all participants in both trials. The participants were asked to refrain from consuming excess alcohol, and to maintain normal eating and sleeping habits during the day before sampling and on the day of sampling itself. Hair was collected over a 24-hour period at 4-hour intervals by firmly holding and pulling the roots of facial hair. The participants were asked to collect hair follicle cells within ±15 minutes at each sampling point and to inform us when they missed a collection. No participants in this study missed any sample collection points. The sampling locations were the laboratory, workplace, and home. In addition, each participant was asked store the samples in the refrigerator immediately after collection, and to then transport the samples to our laboratory on the next day on an ice pack.

### Baseline measurements of anthropometry, diet time, and sleep profile

For each participant, anthropometric variables were established and measured before undertaking the experiments. Body mass and height were measured to the nearest 0.1 kg or cm, respectively, using a digital scale. BMI was calculated as weight in kilograms divided by the square of height in meters.

Participants were asked to respond to questions relating to sleep and diet, such as ‘During the last month, what time did you usually go to sleep and wake up during weekdays and on the weekend?’, ‘On the experimental day, what time did you go to sleep and wake up?’, and ‘What time did you eat breakfast, lunch, and dinner?’.

### Baseline measurements of chronotype and SJL

To assess chronotype, we used the Horne-Ostberg Morningness-Eveningness Questionnaire (MEQ)^11^. The MEQ consists of 19 questions related to preferred sleep time and daily performance (e.g. What would be the best time to perform hard physical work?). The scores ranged from 16 to 86. On the basis of their scores, participants were divided into the following three chronotype groups: morningness (score 59–86), intermediate (score 42–58), or eveningness (score 16–41).

SJL was calculated by subtracting the midpoint of sleep on work or school days from that on a free day^17,20^. The SJ time was calculated from the time of going to sleep and waking up during the weekdays and weekend of the last month. The midpoint of sleep, defined as the time between sleep onset and waking up, was calculated separately for work or school days and the free day. To compare whether SJL had an effect on clock gene expression, we divided the participants into two groups, namely the low SJL group (n = 12) and the high SJL group (n = 12). The high SJL group included participants who had an SJL time greater than 1 hour. This cut-off was selected because it was used in a previous study that examined the relationship between SJL and obesity status and metabolic parameters^23^. The small SJL group included participants who had an SJL time of less than 1 hour.

### Hair follicle cell collection and laboratory assays

Collected hair was rapidly soaked in dissolution buffer (RNeasy Micro Kit; QIAGEN). More than 10 hairs were collected at each sampling point. All samples were stored at −80 °C until RNA purification. An RNeasy Micro Kit (QIAGEN, Hilden, Germany) was used with frozen cytolysis solution to purify total RNA. Total RNA was reverse-transcribed using an Advanced cDNA Synthesis Kit for RT-qPCR (Bio-Rad Laboratories, Inc., California, USA), and real-time PCR was performed using a TaqMan MGB probe (Applied Biosystems, California, USA) and a 1/20 volume of the reverse-transcription product. Data were obtained using a 7500 fast real-time PCR system (Applied Biosystems, California, USA) and expression was normalized to that of 18 S rRNA. The sequences of the primers and probes used are shown in Table 6. These primers and probe were the same as those used in previous studies^28,31^. In the present study, most clock gene expression patterns could not be fitted by 24 h cosine rhythm analysis using the single cosiner method programme (Acro.exe version 3.5)^39^. Therefore, we defined the highest level of clock gene expression throughout the day as the peak time of clock gene expression.

**Table 6:** Primer sequences for real-time RT-PCR analysis and TaqMan MGB probes.

Gene	Forward	Reverse	Probe
*18S*-*rRNA*	CGCCGCTAGAGGTGAAATTC	CGAACCTCCGACTTTCGTTCT	CCGGCGCAAGACGGACCAGA
*PER3*	CTACCTGCACCCTGAAGATCGTTCTC	CTGGAATCCAGTATGATGTAGTCTCCGTTT	CTCTGATGGTTGCCATAC
*NR1D1*	GCTCAGTGCCATGTTCGACTTC	AAGTCTCCAAGGGCCGGTTC	AAGCTCAACTCCCTGGC
*NR1D2*	TCCAGTACAAGAAGTGCCTGAAGAATGAAA	CACGCTTAGGAATACGACCAAACCGA	ATGTCAGCAATGTCG

### Statistical analysis

Data were analysed using Predictive Analytics Software (PASW) version 23.0 for Windows (SPSS Japan Inc., Tokyo, Japan). The Kolmogorov–Smirnov test was used to check for normal marker distribution. The distribution of these markers did not differ significantly from a normal distribution. One-way ANOVA was used to assess differences among groups (morningness, intermediate, and eveningness) at baseline. Paired *t*-tests were used to assess differences between Friday and Monday trials. Un-paired *t*-tests were used to assess differences between the low and high SJL groups. Using a two-way repeated-measures ANOVA, we analysed data for the main effects of trial (Friday and Monday trials), chronotype group (morningness, intermediate, and eveningness), and low and high SJL groups over time (24-hour period), as well as trial × time and group × time interactions. When significant main or interaction effects were detected, we used the Bonferoni method for post-hoc comparisons. One-way ANOVA was used to assess differences in the change from Friday to Monday trials among groups (morningness, intermediate, and eveningness). Unpaired *t*-tests were used to assess differences in the change from Friday to Monday trials between the low SJL and high SJL groups. Partial correlation was used to examine the relationship between MEQ score and SJL time and peak or change in peak time of clock gene expression. Statistical significance was accepted at the 5% level. Results are presented as the mean ± standard error (SE).

## Electronic supplementary material

supplemental data
