# Supplementary material for: Chronotype and social jetlag influence human circadian clock gene expression
Source: Sci Rep. 2018 Jul 5;8:10152. doi: 10.1038/s41598-018-28616-2 (PMC6033857; doi:10.1038/s41598-018-28616-2)
Supplement: Supplementary file 1 — supplemental data [file 41598_2018_28616_MOESM1_ESM.pdf]

# Chronotype and social jetlag influence human circadian clock gene expression

Masaki Takahashi, Yu Tahara, Miku Tsubosaka, Mayuko Fukazawa, Mamiho Ozaki, Tamao Iwakami, Takashi Nakaoka, Shigenobu Shibata

| | Low SJL group (n =12) | High SJL group (n =12) | P value |
|---|---|---|---|
| Age (years) | 27.3 ± 1.1 | 25.8 ± 1.3 | 0.391 |
| Height (cm) | 173.5 ± 1.5 | 172.1 ± 1.4 | 0.482 |
| Weight (kg) | 68.8 ± 1.8 | 67.3 ± 2.8 | 0.667 |
| BMI | 22.8 ± 0.5 | 22.7 ± 0.7 | 0.852 |
| MEQ score | 56.5 ± 2.8 | 42.6 ± 3.3** | 0.004 |
| Wake-up time at weekday (h) | 7.0 ± 0.4 | 7.8 ± 0.4 | 0.142 |
| Bedtime at weekday (h) | 23.9 ± 0.4 | 24.7 ± 0.3 | 0.132 |
| Wake-up time at weekend (h) | 7.7 ± 0.5 | 10.4 ± 0.4* | 0.011 |
| Bedtime at weekend (h) | 24.2 ± 0.4 | 25.6 ± 0.3** | 0.001 |
| Socail jetlag time (h) | 0.5 ± 0.1 | 1.8 ± 0.1** | 0.001 |

Table S1. Physical characteristics of low and high SJL groups.
All data are presented as means ± SE.
P value : un-paired t-test; between groups, *P<0.05, **P<0.01 vs SJL low group

| Breakfast | | *NR1D1* | *NR1D2* | *PER3* |
|---|---|---|---|---|
| Friday | *r* | -0.009 | 0.403 | 0.444 |
| | *p* | 0.974 | 0.109 | 0.074 |
| Monday | *r* | 0.101 | -0.366 | -0.152 |
| | *p* | 0.680 | 0.123 | 0.534 |

| Lunch | | *NR1D1* | *NR1D2* | *PER3* |
|---|---|---|---|---|
| Friday | *r* | 0.108 | 0.271 | 0.032 |
| | *p* | 0.614 | 0.200 | 0.883 |
| Monday | *r* | -0.112 | 0.229 | 0.337 |
| | *p* | 0.601 | 0.282 | 0.107 |

| Dinner | | *NR1D1* | *NR1D2* | *PER3* |
|---|---|---|---|---|
| Friday | *r* | 0.068 | 0.469 | 0.267 |
| | *p* | 0.751 | 0.021 | 0.206 |
| Monday | *r* | 0.410 | 0.536 | 0.576 |
| | *p* | 0.052 | 0.008 | 0.004 |

Table S2. The correlations between peak time of each clock gene and time of breakfast, lunch, and dinner in Friday and Monday trials.
* Breakfast in Friday trials n = 17, breakfast in Monday trials n = 19; * Dinner in Monday trials n = 23
